# Cognitively normal women with Alzheimer’s disease proteinopathy show relative preservation of memory but not of hippocampal volume

**DOI:** 10.1186/s13195-019-0565-1

**Published:** 2019-12-26

**Authors:** Jessica Z. K. Caldwell, Jeffrey L. Cummings, Sarah J. Banks, Sebastian Palmqvist, Oskar Hansson

**Affiliations:** 1 Cleveland Clinic Lou Ruvo Center for Brain Health, 888 W. Bonneville Ave, Las Vegas, NV 89106 USA; 2 UNLV Department of Brain Health, School of Integrated Health Sciences, Box 453019, 4505 S. Maryland Pkwy, Las Vegas, Nevada 89154 USA; 3 University of California, San Diego, 9500 Gilman Drive, La Jolla, CA 92093 USA; 4 Clinical Memory Research Unit, Department of Clinical Sciences in Malmö, Lund University, PO Box 188, 221, Lund, Sweden; 5 Department of Neurology, Skåne University Hospital, 221 85 Lund, Sweden; 6 Memory Clinic, Skåne University Hospital, 205 05 Malmö, Sweden

**Keywords:** Subjective cognitive decline, Mild cognitive impairment, Hippocampus, Sex, Women, Verbal memory

## Abstract

**Background:**

We examined interactive effects of sex, diagnosis, and cerebrospinal fluid (CSF) amyloid beta/phosphorylated tau ratio (Aβ/P-tau) on verbal memory and hippocampal volumes.

**Methods:**

We assessed 682 participants (350 women) from BioFINDER (250 cognitively normal [CN]; and 432 symptomatic: 186 subjective cognitive decline [SCD], 246 mild cognitive impairment [MCI]). General linear models evaluated effects of Alzheimer’s disease (AD) proteinopathy (CSF Aß/p-tau ratio), diagnosis, and sex on verbal memory (ADAS-cog 10-word recall), semantic fluency (animal naming fluency), visuospatial skills (cube copy), processing speed/attention functions (Symbol Digit Modalities Test and Trail Making Part A), and hippocampal volumes.

**Results:**

Amyloid-positive (Aβ/P-tau+) CN women (women with preclinical AD) showed memory equivalent to amyloid-negative (Aβ/P-tau−) CN women. In contrast, Aβ/P-tau+ CN men (men with preclinical AD) showed poorer memory than Aβ/P-tau− CN men. Symptomatic groups showed no sex differences in effect of AD proteinopathy on memory. There was no interactive effect of sex, diagnosis, and Aβ/P-tau on other measures of cognition or on hippocampal volume.

**Conclusions:**

CN women show relatively preserved verbal memory, but not general cognitive reserve or preserved hippocampal volume in the presence of Aβ/P-tau+. Results have implications for diagnosing AD in women, and for clinical trials.

## Background

Memory differs between women and men in ways that may meaningfully impact the detection and course of Alzheimer’s disease (AD). In particular, women have verbal memory strengths that appear to be sustained early in the disease, despite measurable pathological changes, including fluorodeoxyglucose positron emission tomography (FDG PET) abnormalities [1], hippocampal volume (HV) loss [2], and amyloid beta (Aβ) protein accumulation as shown by a positive amyloid PET scan [3, 4]. This early preserved memory may delay diagnosis [5], which is troubling given evidence that women—and particularly those with risk factors such as Apolipoprotein ε4 (*APOE* ε*4*) genotype—may decline faster than men, once AD-related cognitive decline has begun [6–9]. The exact timing of women’s memory changes in AD remains unknown and a topic of interest [10].

Memory advantages in women throughout the lifespan could relate to a variety of etiological factors (e.g., [9, 11–14]). Proximally, preservation of memory is expected to be reflected in resilience in neural structures and functions that subserve healthy memory. Although memory relies on a complex network of neural regions interacting effectively, the central role of the hippocampus in episodic memory (for a review, see [15]) and in AD [16] makes this structure a natural candidate for underlying neural resilience. Specifically, the hippocampus is critical for memory consolidation, and damage to the hippocampus can lead to decreased ability to learn and recall new information [15]. In AD, the hippocampus is impacted by pathology early in the disease course, with neurofibrillary tangle buildup beginning in the cornu ammonis 1 (CA1) and subiculum regions and progressing throughout the hippocampus in a predictable fashion [16].

Previous work from our group has built preliminary support for relatively preserved total and right hippocampal volume (HV) [3] and right subiculum subfield volume [17] in women. Specifically, with a trend-level finding, our data suggested that sex moderated the effects of diagnosis and amyloid PET positivity on HV. In women, positive amyloid PET related to smaller HV only at the mild cognitive impairment (MCI) stage, but not in cognitively normal women. The pattern in men was similar, but the differences were not significant [3]. When we examined these findings by hippocampal subfield, we replicated the effect only within the right subiculum [17].

Importantly, if sex relates to a time-limited preservation of memory and/or HV in AD, analyses of sex effects should consider both diagnostic trajectory (i.e., normal cognition to MCI to dementia) and presence of biomarkers such as brain amyloid and tau measured by PET or indexed by the cerebrospinal fluid (CSF). This process allows for higher certainty that sex effects are occurring in AD and not general to non-AD cognitive impairment.

Work on sex differences in AD has been done with large, well-characterized study samples, and especially in one study population (the Alzheimer’s Disease Neuroimaging Initiative [ADNI]). However, a key step in understanding sex differences in AD is exploring whether findings within ADNI replicate in different samples and with different measures.

The current investigation sought to examine whether the effects of AD proteinopathy on memory and hippocampal volume differ based on sex in the Swedish BioFINDER cohort. BioFINDER offers advantages in that it includes a clinically representative sample of patients who were consecutively referred to participating memory clinics and control participants recruited from an ongoing population-based study (the Malmö Diet and Cancer study) [18]. Both groups on average have levels of education more typical of an aging population than in some other large study samples [19] and were scanned on the same magnetic resonance (MR) scanner. BioFINDER also includes measures of other cognitive domains, including visuospatial skills, attention, and processing speed skills, allowing for assessment of the specificity of effects to the memory domain. Altogether, these advantages position the BioFINDER [20] sample well to identify associations between biomarkers of pathological change and cognition.

Based on our prior results [3, 17], we hypothesized that women would show early relative preservation of memory and HV, such that these variables would be impacted less by AD proteinopathy (Aβ/P-tau+) in CN women than in CN men. In other words, women, but not men, with preclinical AD would show early preservation of these variables. We hypothesized this effect would be specific to memory and not generalized to other cognitive domains. We further hypothesized that at the symptomatic stage, Aβ+ women would no longer show relative preservation of memory and HV, and no sex differences would be observed.

## Methods

### Participants

Participants were selected from the Swedish BioFINDER study, which is a prospective, longitudinal study examining disease mechanisms in AD and other neurodegenerative disorders using several fluid and imaging biomarkers (see http://biofinder.se for more information about the study design). For the present study, we included participants with CSF and MRI data from the healthy elderly control cohort (*n* = 250) and all non-demented patients that had been referred to participating memory clinics due to cognitive symptoms (*n* = 432).

The CN elderly participants were consecutively enrolled from a population-based cohort in the South of Sweden (Malmö Diet and Cancer Study [18]). The inclusion criteria were (1) age ≥ 60 years old, (2) Mini-Mental State Examination (MMSE) [21] score of 28–30 points, and (3) fluent in Swedish. The exclusion criteria were (1) presence of subjective cognitive impairment, (2) significant neurologic disease (for example, stroke, Parkinson’s disease, multiple sclerosis), (3) severe psychiatric disease (for example, severe depression or psychotic syndromes), and (4) dementia or mild cognitive impairment (MCI).

The patients with cognitive symptoms (“symptomatic patients”) had all been referred to one of three participating memory clinics in the south of Sweden, mostly from primary care, and consecutively enrolled in BioFINDER based on the following inclusion and exclusion criteria: (1) perceived cognitive decline, (2) did not fulfill Diagnostic and Statistical Manual of Mental Disorders, 5th Edition (DSM-5) [22] criteria for Major Neurocognitive Disorder (dementia), as assessed by a memory clinic physician, (3) had a MMSE score of 24 to 30 points, (4) were aged 60 to 80 years, and (5) were fluent in Swedish. The exclusion criteria were (1) cognitive impairment that could be explained by another condition (other than prodromal dementia), such as brain tumor, (2) severe somatic disease, and (3) refusing lumbar puncture or neuropsychological testing.

The patients were further categorized as having subjective cognitive decline (SCD) (*n* = 186) or MCI (*n* = 246). The MCI classification was based on the results of a comprehensive neuropsychological battery and the clinical assessment of a senior neuropsychologist and two physicians [23]. Patients with composite *z*-scores of ≤ − 1.5 standard deviations (SD) in at least one cognitive domain were classified as MCI (at least two different tests were used for each cognitive domain). In agreement with the DSM-5 criteria for mild neurocognitive disorders, all subjects with *z*-scores of − 1 to − 1.5 were individually assessed by the neuropsychologist and classified as MCI if their premorbid ability or individual test scores within each domain indicated a significant cognitive decline. Among the MCI participants, 75% were categorized as amnestic MCI and 25% as non-amnestic MCI. The participants with cognitive complaints who did not fulfill the criteria for MCI or dementia were classified as having SCD. Neuropsychological test measures incorporated in the following statistical analyses were not included in the battery used to determine diagnosis.

The study was approved by the ethical review board in Lund, Sweden, and all participants gave their written informed consent.

### CSF analysis and classification of Aβ/P-tau status

Lumbar puncture (LP) and CSF procedures followed a previously described protocol [22]. CSF Aβ42 and phosphorylated tau (P-tau) were analyzed using the Elecsys immunoassays on a cobas e601 analyzer at the Clinical Neurochemistry Laboratory, University of Gothenburg, Sweden.

Aβ/P-tau positivity was defined based on a previously defined CSF cut-off (phosphorylated tau/Aβ42 ratio ≥ 0.022). This cutoff has been validated against FDA-approved visual reads of Aβ PET scans with 90% agreement [24].

### MRI procedures and hippocampal volume

All participants were examined using the same MR scanner (3 Tesla Siemens Tim Trio). The MR scanning and imaging procedures have been described previously [25]. FreeSurfer software (version 5.3) was used to extract data for the total intracranial volume and total HV (left and right).

### Memory function

The 10-word list from the Alzheimer’s Disease Assessment Scale – cognitive subscale (ADAS-cog) was used to test memory function [26]. After three learning trials of 10 words (immediate recall) and a distraction task (naming objects and fingers), the participants were asked to recall the 10 words. The number of omissions on the delayed recall task (i.e., total possible recalled—total recalled) constituted the final test score. Commission errors were not analyzed.

### Non-memory functions

Semantic fluency was assessed using the animal naming fluency test [27]. Visuospatial function was assessed with a cube copying task [28] scored from 0 to 6 points [26]. Attention and speed were assessed using Trail Making Test Part A (TMT A) [29] and the Symbol Digit Modalities Test (SDMT) [30].

### Statistical analysis

Groups were compared using the Mann-Whitney Test. A *p* value of < 0.05 was used to define statistical significance. Several general linear models (GLMs) were used to test the effect of sex on cognitive performance in the presence of AD proteinopathy at different diagnostic stages. First, we examined memory function (delayed recall omissions) as the dependent variable with the independent variables Aβ/P-tau status, diagnostic group, sex, years of formal education, presence of at least one *APOE* ε4 allele, HV, and total intracranial volume. Interaction effects were tested for Aβ/P-tau status, diagnostic group, and sex. This model was also run using animal naming fluency, cube copying, TMT A, and SDMT as dependent variables. Diagnostic group was primarily stratified into CN and symptomatic patients to achieve better statistical power for the primary analyses; in secondary analyses, the symptomatic patients were further stratified into SCD and MCI patients. To further examine sex differences in the effect of AD proteinopathy on memory, we ran GLMs using delayed recall omissions as dependent variable and Aβ/P-tau, age, and education as independent variables. This model was tested separately for men and women in all diagnostic subgroups (primary analyses separately in CN and symptomatic patients, and in secondary analyses with symptomatic patients stratified into SCD and MCI). Here, (Aβ/P-tau+) CN women are referred to as women with preclinical AD, and (Aβ/P-tau+) CN men are similarly referred to as men with preclinical AD.

Next, sex differences in the effect of AD proteinopathy on HV were examined in the different diagnostic groups. Here, we used models with HV as a dependent variable with the independent variables Aβ/P-tau, sex, diagnostic group, age, education, presence of at least one *APOE* ε4 allele, and total intracranial volume. Total HV, left HV, and right HV were used separately as dependent variables. As for the GLMs described above, interaction effects were tested for Aβ/P-tau status, diagnostic group, and sex (as above, diagnostic group was stratified both as CN or symptomatic patient and, in secondary analyses as CN, SCD, or MCI). In the secondary analyses where symptomatic patients were further stratified into SCD and MCI, interaction effects were analyzed with Mann-Whitney tests due to smaller sample sizes and skewed distributions. All statistical analysis was performed using R version 3.4.4 (The R Foundation for Statistical Computing).

## Results

### Demographics

Of 682 participants, 350 were women, 241 were Aβ/P-tau+, and 267 were *APOE* ε4 carriers. Regarding diagnosis, 250 were CN and 432 had cognitive symptoms (of which 186 had subjective and 246 objective symptoms). Average age was 71.7 (SD = 5.5). Of CN participants, 152 were women, 67 were *APOE* ε4 carriers (39 women), and 45 were Aβ/P-tau+ (29 women). Of symptomatic patients, 198 were women, 200 were *APOE* ε4carriers (90 women), and 196 were Aβ/P-tau+ (88 women). See Tables 1 and 2 for additional demographic and descriptive information.

**Table 1:** Means and standard deviations by diagnostic group (cognitively normal or symptomatic), sex, and Aβ/P-tau status for demographics; memory and global cognitive scores, hippocampal and total intracranial volumes; and number of APOE ε4 carriers

	Cognitively normal (CN)				Symptomatic patients				
	Men		Women		Men			Women	
	Aβ/P-tau+	Aβ/P-tau−	Aβ/P-tau+	Aβ/P-tau−	Aβ/P-tau+	Aβ/P-tau−		Aβ/P-tau+	Aβ/P-tau−
	(*N* = 16)	(*N* = 82)	(*N* = 29)	(*N* = 123)	(*N* = 108)	(*N* = 126)		(*N* = 88)	(*N* = 110)
Age	74.38 (4.40) ^b^	72.40 (4.58) ^b^	74.38 (4.34) ^b^	74.03 (5.31) ^b^	72.43 (5.18) ^a,b^	69.84 (5.61) ^a,b^		71.12 (5.05) ^a,b^	69.08 (5.68) ^a,b^
Education	14.12 (4.29)	12.17 (3.58)	11.97 (4.23)	12.08 (3.30)	11.65 (3.58)	11.63 (3.69)		11.51 (3.31)	12.18 (3.28)
ADAS Delayed Word Recall Omissions (/10)	3.31 (2.39)	2.12 (1.63)	2.07 (2.17)	1.74 (1.91)	6.00 (2.39)	4.90 (2.37)		6.50 (2.40)	3.50 (2.46)
MMSE total score	28.69 (0.87)	29.09 (0.98)	29.17 (0.76)	28.91 (0.99)	27.19 (1.80)	28.11 (1.78)		27.1 (1.78)	28.31 (1.57)
Total intracranial volume	1.7 × 10^6^ (1.5 × 10^5^)	1.7 × 10^6^ (1.3 × 10^5^)	1.5 × 10^6^ (9.7 × 10^4^)	1.5 × 10^6^ (1.1 × 10^5^)	1.7 × 10^6^ (1.2 × 10^5^)	1.7 × 10^6^ (1.4 × 10^5^)		1.5 × 10^6^ (1.2 × 10^5^)	1.5 × 10^6^ (1.2 × 10^5^)
Total hippocampal volume	7.8 × 10^3^ (1.0 × 10^3^)	7.7 × 10^3^ (1.1 × 10^3^)	7.0 × 10^3^ (8.9 × 10^2^)	7.1 × 10^3^ (9.1 × 10^2^)	6.8 × 10^3^ (1.1 × 10^3^)	7.4 × 10^3^ (1.3 × 10^3^)		6.3 × 10^3^ (1.1 × 10^3^)	7.1 × 10^3^ (1.1 × 10^3^)
*APOE* ε4 carriers	12 ^a^	16 ^a^	16 ^a^	23 ^a^	76 ^a^	34 ^a^		63 ^a^	27 ^a^

*Abbreviations*: *Aβ/P-tau+* amyloid beta/P-tau positive, *ADAS* Alzheimer’s Disease Assessment Scale, *APOE* apolipoprotein E, *MMSE* Mini-Mental State Examination

^a^Differs by Aβ/P-tau+ within diagnostic group, *p* < 0.001

^b^Differs by sex within diagnostic group, *p* < 0.05

Significant differences in these variables by Aβ/P-tau status and sex within each diagnostic group are indicated by superscript

**Table 2:** Means and standard deviations by symptomatic diagnostic group (subjective cognitive decline or mild cognitive impairment), sex, and Aβ/P-tau status for demographics; memory and global cognitive scores, hippocampal and total intracranial volumes; and number of APOE ε4 carriers

	Subjective cognitive decline (SCD)				Mild cognitive impairment (MCI)			
	Men		Women		Men		Women	
	Aβ/P-tau+	Aβ/P-tau−	Aβ/P-tau+	Aβ/P-tau−	Aβ/P-tau+	Aβ/P-tau−	Aβ/P-tau+	Aβ/P-tau−
	(*N* = 34)	(*N* = 51)	(N = 25)	(*N* = 76)	(*N* = 74)	(*N* = 75)	(*N* = 63)	(*N* = 34)
Age	72.59 (5.37) ^a,b^	70.22 (5.46) ^a,b^	70.63 (4.84) ^a,b^	69.78 (5.69) ^a,b^	72.35 (5.13) ^a^	69.59 (5.74) ^a^	71.37 (4.88) ^a^	69.76 (5.52) ^a^
Education	11.71 (3.84) ^a^	13.00 (3.59) ^a^	11.60 (3.33) ^a^	11.97 (3.30) ^a^	11.62 (3.49) ^a^	10.71 (3.48) ^a^	11.44 (3.07) ^a^	10.47 (3.13) ^a^
ADAS Delayed Word Recall Omissions (/10)	4.12 (1.89)	3.38 (1.89)	5.06 (3.02)	4.73 (2.80)	6.91 (2.06) ^a^	5.92 (2.10) ^a^	7.43 (1.81) ^a^	5.24 (2.69) ^a^
MMSE total score	28.35 (1.54)	28.92 (1.29)	27.88 (1.88)	27.74 (1.73)	26.65 (1.67)	27.56 (1.85)	26.84 (1.79)	27.62 (1.88)
Total intracranial volume	1.7 × 10^6^ (1.1 × 10^5^)	1.7 × 10^6^ (1.3 × 10^5^)	1.5 × 10^6^ (1.1 × 10^5^)	1.5 × 10^6^ (1.2 × 10^5^)	1.7 × 10^6^ (1.3 × 10^5^)	1.7 × 10^6^ (1.4 × 10^5^)	1.5 × 10^6^ (1.2 × 10^5^)	1.5 × 10^6^ (1.2 × 10^5^)
Total hippocampal volume	7.2 × 10^3^ (1.1 × 10^3^)	7.7 × 10^3^ (1.3 × 10^3^)	6.8 × 10^3^ (1.3 × 10^3^)	6.7 × 10^3^ (1.1 × 10^3^)	6.7 × 10^3^ (1.1 × 10^3^)	7.2 × 10^3^ (1.3 × 10^3^)	6.1 × 10^3^ (1.0 × 10^3^)	6.6 × 10^3^ (1.2 × 10^3^)
*APOE* ε4 carriers	21 ^a^	14 ^a^	17 ^a^	21 ^a^	55 ^a^	20 ^a^	46 ^a^	6 ^a^

*Abbreviations*: *Aβ/P-tau+* amyloid beta/P-tau positive, *ADAS* Alzheimer’s Disease Assessment Scale, *APOE* apolipoprotein E, *MMSE* Mini-Mental State Examination

^a^Differs by Aβ/P-tau+ within diagnostic group, *p* < 0.05

^b^Differs by sex within diagnostic group, *p* < 0.05

Significant differences in these variables by Aβ/P-tau status and sex within each diagnostic group are indicated by superscript

Among CN participants, Mann-Whitney tests showed that age and education did not differ by amyloid status (age: *W* = 3955.5, *p* = 0.13; education: *W = 4433.0, p* = 0.68), but a greater percentage of Aβ/P-tau+ versus Aβ/P-tau− CN individuals were *APOE* ε4 carriers (*W =* 2482.0, *p* < 0.001). Mann-Whitney tests also revealed CN women were older (*W =* 6439.5, *p* = 0.05), but CN men and women did not differ in education level (*W =* 7969.0, *p* = 0.35) or number of *APOE* ε4 carriers (*W =* 7490.0, *p* = 0.57) (see Table 1).

Within symptomatic participants, Mann-Whitney tests showed that Aβ/P-tau+ individuals were older (*W =* 17,639.0, *p* < 0.001) and were more likely to be *APOE* ε4 carriers (*W =* 12,647.0, *p* < 0.001). Symptomatic Aβ/P-tau+ versus Aβ/P-tau− individuals did not differ in education level (*W =* 23,862.0, *p* = 0.36). Mann-Whitney tests also revealed men and women with cognitive symptoms did not differ in education level (*W =* 21,386.0, *p* = 0.30) or number of *APOE* ε4 carriers (*W =* 23,418.0, *p* = 0.69), but men were older (*W =* 25,686.0, *p* = 0.05) (see Table 1).

Secondary Mann-Whitney analyses examining differences within SCD and MCI individuals showed that within SCD, Aβ/P-tau+ individuals were older (*W =* 2852.5, *p* < 0.01), more likely to be *APOE* ε4 carriers (*W =* 2345.0, *p* < 0.001), and had lower education levels (*W =* 4576.5, *p* = 0.015). Mann-Whitney tests revealed men and women with SCD did not differ in education level (*W =* 414,105.0, *p* = 0.68) or number of *APOE* ε4 carriers (*W =* 4382.00, *p* = 0.53), but men were older (*W =* 5094.5, *p* = 0.03). For MCI individuals, Aβ/P-tau+ individuals were older (*W =* 5778.0, *p* < 0.01), more likely to be *APOE* ε4 carriers (*W =* 3743.0, *p* < 0.001), and had higher education levels (*W =* 6019.5, *p* = 0.03). Mann-Whitney tests showed men and women with SCD did not differ in education level (*W =* 6814.0, *p* = 0.83), number of *APOE* ε4 carriers (*W =* 6990.0, *p* = 0.62), or age (*W =* 7455.5, *p* = 0.67) (see Table 2).

### Interactive effects of sex, diagnosis, and amyloid status on delayed verbal recall

The GLM with sex, diagnosis (CN or symptomatic), Aβ/P-tau+, and their interactions predicting delayed recall omissions on the ADAS word recall task showed a significant three-way interaction of Aβ/P-tau+, diagnosis, and sex (*p* < 0.001) as well as a two-way interaction of Aβ/P-tau+ and sex (*p* = 0.008). These interactions were observed in a model including age, education, *APOE* ε4 status, and total HV as covariates (Table 3). A secondary analysis that grouped diagnoses as CN, SCD, or MCI showed a similar three-way interaction of Aβ/P-tau+, diagnosis, and sex (*p* = 0.01; see Table 4).

**Table 3:** Results for regression models with sex, diagnosis (cognitively normal or symptomatic), Aβ/P-tau status, and their interactions predicting recall omissions on the ADAS word recall task and total hippocampal volume

Variable	ADAS 10 Word Recall Omissions	
	Estimate	*p*
Intercept	0.56	0.704
Diagnosis (0 = CN, 1 = symptomatic)	2.51	< 0.001
Age	0.0088	0.61
Education	−0.107	< 0.001
Total intracranial volume (*z*-scored)	0.225	0.039
Sex (0 = male)	0.22	0.74
Hippocampal volume (*z*-scored)	−0.781	< 0.001
APOE ε4 Genotype (0 = no ε4)	0.049	0.79
CSF Aβ/P-tau positivity (Aβ/P-tau+) (0 = not Aβ/P-tau+)	1.97	0.10
Aβ/P-tau+ × diagnosis	−0.63	0.33
Aβ/P-tau+ × sex	−4.05	0.008
Diagnosis × sex	−0.76	0.06
Aβ/P-tau+ × diagnosis × sex	2.92	< 0.001
Variable	Total hippocampal volume	
	Estimate	*p*
Intercept	10,938	< 0.001
Diagnosis (0 = CN, 1 = symptomatic)	− 547	< 0.001
Age	−82.8	< 0.001
Education	5.00	0.64
Total intracranial volume (*z*-scored)	0.0019	< 0.001
Sex (0 = male)	− 166	0.59
APOE ε4 Genotype (0 = no ε4)	20.7	0.81
Aβ/P-tau+ (0 = not Aβ/P-tau+)	453	0.42
Aβ/P-tau+ × diagnosis	− 401	0.18
Aβ/P-tau+ × sex	−72.1	0.92
Diagnosis × sex	77.8	0.68
Aβ/P-tau+ × diagnosis × sex	−76.0	0.84

Abbreviations: *Aβ/P-tau+* amyloid beta/P-tau positivity, *ADAS* Alzheimer’s disease Assessment Scale, *APOE* apolipoprotein E, *CN* cognitively normal, *CSF* cerebrospinal fluid, *MMSE* Mini-Mental State Examination

significant p values had been bolded (*p* < .05)

**Table 4:** Results for secondary regression model with sex, diagnosis (cognitively normal, subjective cognitive decline, or mild cognitive impairment), Aβ/Ptau status, and their interactions predicting recall omissions on the ADAS word recall task

Variable	ADAS 10 Word Recall Omissions	
	Estimate (CI)	*p*
Intercept	−.986 (−2.14–4.90)	0.454
Diagnosis (1 = CN, 2 = SCD, 3 = MCI)	1.75 (1.44–2.07)	*< 0.001*
Age	0.03 (−0.00–0.60)	*0.035*
Education	−0.07 (−0.12 to − 0.03)	*< 0.001*
Total intracranial volume (*z*-scored)	0.081 (−0.12–0.28)	0.419
Sex (0 = male)	−0.40 (−1.31–0.51)	0.392
Hippocampal volume (*z*-scored)	− 0.517 (− 0.70 to − 0.34)	*< 0.001*
*APOE* ε4 genotype (0 = no ε4)	0.01 (− 0.33–0.35)	0.96
CSF Aβ/P-tau positivity (Aβ/P-tau+) (0 = not Aβ/P-tau+)	0.64 (− 0.77–2.04)	0.376
Aβ/P-tau+ × diagnosis	0.03 (− 0.54–0.60)	0.92
Aβ/P-tau+ × sex	− 1.50 (− 3.32–0.32)	0.108
Diagnosis × sex	−0.20 (− 0.65–0.26)	0.398
Aβ/P-tau+ × diagnosis × sex	1.03 (0.25–1.82)	*0.01*

Note: Estimates, confidence intervals, and *p* values were calculated for each regression model

Abbreviations: *Aβ/P-tau+* amyloid beta/P-tau positive, *ADAS* Alzheimer’s Disease Assessment Scale, *APOE* apolipoprotein E, *CSF* cerebrospinal fluid, *MMSE* Mini-Mental State Examination

**Fig. 1:**
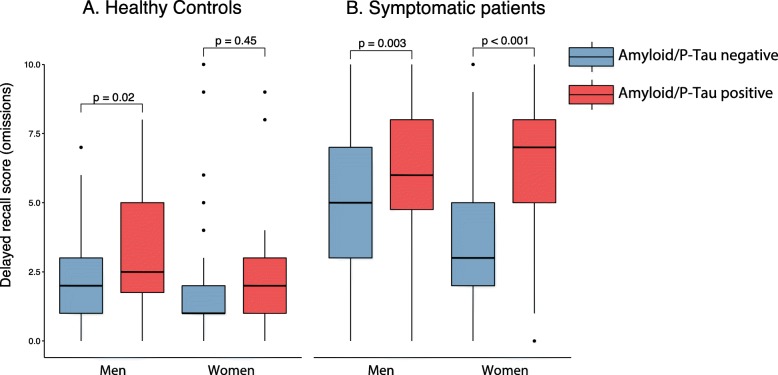
Comparison of the Aβ/P-tau effect on memory in men and women. Comparison of 10 word delayed recall performance in Aβ/P-tau+ and Aβ/P-tau− participants, stratified by sex, in **a** healthy controls and **b** patients with cognitive symptoms. Comparisons were adjusted for age and education. Further comparison among the symptomatic patients (SCD and MCI) is shown in Additional file 1: Figure S1

Parsing this interaction effect indicated that women but not men showed a significant interaction between amyloid status and diagnosis (subsample with men, *p* = 0.42; subsample with women, *p* < 0.001). Specifically, Aβ/P-tau+ did not impact delayed recall memory in women in the CN group (*p* = 0.45, adjusted for age and education), but related to poorer memory in symptomatic women (*p* < 0.001). In contrast, Aβ/P-tau+ was associated with poorer memory performance both in CN men (*p* = 0.02) and in symptomatic men (*p* = 0.003) adjusted for age and education (Fig. 1). A secondary analysis, splitting the symptomatic group into SCD and MCI, showed Aβ/P-tau+ related to poorer memory both in female SCD (*p* = 0.02) and MCI (*p* < 0.001) and in male SCD (*p* = 0.057) and MCI (*p* = 0.006) (see Additional file 1: Figure S1).

### Interactive effects of sex, diagnosis, and amyloid status on verbal semantic fluency, visuospatial function, and attention/processing speed

The above models with sex, diagnosis (CN or patients with cognitive symptoms), Aβ/P-tau+, and their interactions were also tested for predicting performance on verbal semantic fluency (animal naming fluency), visuospatial function (cube copying), and attention/processing speed (TMT A and SDMT). The models were, as for memory function, adjusted for age, education, *APOE* ε4 status, and total HV. In contrast to using memory performance as outcome, the three-way interaction of Aβ/P-tau+, diagnosis, and sex was not significant for verbal fluency (*p* = 0.11), visuospatial function (*p* = 0.60), or attention/processing speed (TMT A, *p* = 0.86; SDMT, *p* = 0.96).

### Interactive effects of sex, diagnosis, and amyloid status on hippocampal volume

In contrast to the memory analysis, the GLM with sex, diagnosis, Aβ/P-tau+, and their interactions predicting total HV did not show a significant interaction of Aβ/P-tau+, diagnosis, and sex (*p* = 0.91). However, diagnosis, age, and total intracranial volume (ICV) showed significant main effects on HV. Specifically, older age was associated with smaller HV (*p* < 0.001), as was having cognitive symptoms (*p* < 0.001). Greater total intracranial volume was associated with greater HV (*p* < 0.001) (see Table 3 for details). Similar significant main effects and similar lack of interaction effects were seen when grouping diagnoses into CN, SCD, and MCI and when left and right HV were examined separately (interaction: left: *p* = 0.68; right: *p* = 0.85; total *p* = 0.91) (see Additional file 2 for details).

## Discussion

The current study showed that women with AD proteinopathy (Aβ/P-tau+) showed no memory impairment relative to Aβ/P-tau− women prior to self-reporting concerns about their cognition (i.e., only in the CN group). In contrast, men with Aβ/P-tau+ performed more poorly on the verbal memory task than Aβ/P-tau− men, regardless of whether they experienced cognitive symptoms or not. As hypothesized, similar effects were not seen on tests of other cognitive domains. Analyses examining HV showed no significant interactive effects of sex, diagnosis, and Aβ/P-tau+, and diagnosis alone of these three factors showed a significant main effect on HV.

These results supported our hypotheses about sex-based preservation of verbal memory. Namely, Aβ/P-tau+ CN women (i.e., women with preclinical AD) appear to have verbal memory reserve or resilience in the presence of measurable AD-related disease burden. This result is consistent with our and others’ work showing that women’s memory has some early resilience to a number of markers of AD burden, including abnormal brain metabolism [1], hippocampal atrophy [2], and positive amyloid PET [3, 4]. Importantly, the current results replicate our prior findings in a separate sample that incorporated different measures of memory and amyloid proteinopathy, supporting the robustness of this finding [3]. Early verbal memory preservation may have implications for how normal cognition is defined in research and in clinical trials. For example, it may be more critical when examining longitudinal or interventional outcomes to define baseline group membership using biomarkers in combination with cognitive testing, if women are included in the sample. From a clinical diagnosis perspective, this finding emphasizes the importance of cognitive baseline assessment, as early neuropsychological testing may be able to identify women who have normal cognition yet have lost some memory functioning over time. Employing memory assessments that are not purely verbal may also be important for increasing validity of memory assessment across sexes.

Supplementary analyses showed that verbal memory was not preserved in Aβ/P-tau+ women with SCD as compared to Aβ/P-tau− women with SCD. In addition, in the SCD group, women no longer outperformed men in verbal memory. This pattern suggests that among women with increased risk for AD dementia (Aβ/P-tau+), memory reserve or resilience is limited to CN women with no cognitive concerns. This finding is consistent with studies showing that SCD is associated with positive AD biomarkers and longitudinal cognitive decline ([for recent review on SCD, see [31]), as well as with research showing that AD proteinopathy in the context of SCD is a strong predictor of decline [32]. However, this finding stands in contrast to research within the ADNI sample, showing that women outperform men on verbal memory tasks despite mild to moderate levels of AD-related burden [1, 2]. It is possible that the current finding relates to Aβ/P-tau+ women with SCD in the BioFINDER sample having more than a moderate level of AD-related disease burden. Alternatively, the finding may relate to other differences between samples. Specifically, the greater education levels—and thereby greater general cognitive reserve—in the ADNI sample may have an impact. On the other hand, in the BioFINDER sample, individuals with SCD have been referred to a memory specialist due to cognitive symptoms, whereas in ADNI, individuals with SCD reported symptoms when queried. In this context, our findings suggest that for women seeking a memory specialist, SCD could be not only a marker of risk, but also an indicator that subclinical memory changes are already measurable on cognitive testing. This suggestion is in part consistent with very recent work in other cohorts [33, 34].

The current study did not confirm hypotheses about sex-specific HV preservation. Previously, we have shown in the ADNI sample that CN women with positive amyloid PET studies show no difference in total HV and subiculum subfield volume, compared to CN women with negative amyloid PET, but that volumetric decrements are observed in amyloid-positive women at the MCI stage [3, 17]. Reasons for lack of replication are unclear but could include slight differences in methodology or sample composition, such as inclusion of CN and symptomatic groups in the present analysis as well as differences in the biomarkers used to define the presence of proteinopathy. Lack of replication emphasizes the need for additional research on how this structure—with known developmental [35], aging-related [36, 37], and neurochemical sex differences [38]—does or does not show patterns of dysfunction and atrophy that differ by sex in AD. In addition, this finding underscores the importance of considering other neural underpinnings of early sex-based memory preservation and later decline.

As important context, there are known sex by *APOE* ε4 interactive effects, with evidence for more deleterious effects of *APOE* ε4 on cognition, hippocampal structure, brain function at rest, and tau pathology in women than men ([6, 39], for a recent review see [40]). The present analysis showed that men and women did not differ in number of *APOE* ε4 carriers and also controlled for effects of *APOE* ε4. Despite these efforts to show our findings were not driven by *APOE* ε4 status, adding *APOE* ε4 as an interaction term was beyond the scope of the present analysis due to limited power. Further work is needed on how memory reserve presents over the AD spectrum in women with *APOE* ε4.

Limitations of this analysis include having a smaller sample size than studies that combine across cohorts, which is particularly relevant when evaluating complex interactions. Although the current study in part replicates prior work by our group [3], the challenges of complex interactions and multiple comparisons mean that wider replication is important to ensure generalizable conclusions. Expanding the analysis to additional regions of interest will also be important for generalizability. The sample included is also majority white, and research in diverse samples will be key to generalizing the findings to all women. The current analysis was also cross-sectional in nature, limiting ability to interpret memory findings as true losses of function over time. In contrast, the present study has strengths in that participants are more representative of the general aging population than in some other cohorts [18, 19], were more thoroughly assessed and diagnosed, and have had brain imaging conducted on the same MRI magnet.

## Conclusions

In conclusion, this study shows relative preservation of verbal memory in the presence of AD proteinopathy, limited to women with normal cognition, and not in women with reported or measured memory symptoms. This resilience was specific to memory and was not present for other cognitive functions. Future studies should examine other potential neural sources of sex-based early memory preservation, conduct additional multi-cohort analyses of complex sex-based interactive effects—including longitudinally—and examine the practical effects of sex differences in memory on clinical diagnosis and clinical trial inclusion and outcomes.

## Supplementary information

**Additional file 1:** **Figure S1.** Comparison of the Aβ/P-tau effect on memory in men and women. Comparison of 10 word delayed recall performance in Aβ/P-tau+ and Aβ/P-tau- participants, stratified by sex, in patients with A. subjective cognitive decline, and B. mild cognitive symptoms.

**Additional file 2:** Regression Predicting Hippocampal Volumes with Cognitively Normal, Subjective Cognitive Decline, and Mild Cognitive Impairment Groups. This additional file contains a table summarizing the results of a regression predicting hippocampal volumes separated by diagnostic group.

## Data Availability

The datasets used and/or analyzed during the current study are available from authors Sebastian Palmqvist and Oskar Hansson on reasonable request.

## Abbreviations

AD: Alzheimer’s disease
ADAS-Cog: Alzheimer’s Disease Assessment Scale – cognition
ADNI: Alzheimer’s Disease Neuroimaging Initiative
Aβ: Amyloid beta
CN: Cognitively normal
CSF: Cerebrospinal fluid
FDG PET: Fluorodeoxyglucose positron emission tomography
GLM: General linear models
ICV: Total intracranial volume
LP: Lumbar puncture
MCI: Mild cognitive impairment
MMSE: Mini-Mental State Examination
MR: Magnetic resonance
P-tau: Phosphorylated Tau
SCD: Subjective cognitive decline

## References

[1] Sundermann Erin E., Maki Pauline M., Rubin Leah H., Lipton Richard B., Landau Susan, Biegon Anat (2016). Female advantage in verbal memory. Neurology.

[2] Sundermann Erin E., Biegon Anat, Rubin Leah H., Lipton Richard B., Mowrey Wenzhu, Landau Susan, Maki Pauline M. (2016). Better verbal memory in women than men in MCI despite similar levels of hippocampal atrophy. Neurology.

[3] Caldwell JZK, Berg J-L, Cummings JL, Banks SJ. Moderating effects of sex on the impact of diagnosis and amyloid positivity on verbal memory and hippocampal volume. Alzheimers Res Ther. 2017. 10.1186/s13195-017-0300-8. 10.1186/s13195-017-0300-8 PMC5596932 28899422

[4] Sundermann EE, Biegon A, Rubin LH, Lipton RB, Landau S, Maki PM (2017). Does the female advantage in verbal memory contribute to underestimating Alzheimer’s disease pathology in women versus men?. J Alzheimers Dis.

[5] Brunet H, Caldwell JZK, Brandt J, Miller JB. Influence of sex differences in interpreting learning and memory within a clinical sample of older adults. Aging Neuropsychol Cogn. 2019. 10.1080/13825585.2019.1566433. 10.1080/13825585.2019.1566433 PMC6677625 30663493

[6] Buckley Rachel F., Mormino Elizabeth C., Amariglio Rebecca E., Properzi Michael J., Rabin Jennifer S., Lim Yen Ying, Papp Kathryn V., Jacobs Heidi I.L., Burnham Samantha, Hanseeuw Bernard J., Doré Vincent, Dobson Annette, Masters Colin L., Waller Michael, Rowe Christopher C., Maruff Paul, Donohue Michael C., Rentz Dorene M., Kirn Dylan, Hedden Trey, Chhatwal Jasmeer, Schultz Aaron P., Johnson Keith A., Villemagne Victor L., Sperling Reisa A. (2018). Sex, amyloid, and APOE ε4 and risk of cognitive decline in preclinical Alzheimer's disease: Findings from three well-characterized cohorts. Alzheimer's & Dementia.

[7] Sohn D, Shpanskaya K, Lucas JE, Petrella JR, Saykin AJ, Tanzi RE, et al. Sex differences in cognitive decline in subjects with high likelihood of mild cognitive impairment due to Alzheimer’s disease. Sci Rep. 2018. 10.1038/s41598-018-25377-w. 10.1038/s41598-018-25377-w PMC5945611 29748598

[8] Burke Shanna L., Hu Tianyan, Fava Nicole M., Li Tan, Rodriguez Miriam J., Schuldiner Katie L., Burgess Aaron, Laird Angela (2018). Sex differences in the development of mild cognitive impairment and probable Alzheimer’s disease as predicted by hippocampal volume or white matter hyperintensities. Journal of Women & Aging.

[9] Goodman Y, Bruce AJ, Cheng B, Mattson MP (1996). Estrogens attenuate and corticosterone exacerbates excitotoxicity, oxidative injury, and amyloid-peptide toxicity in hippocampal neurons. J Neurochem.

[10] Nebel Rebecca A., Aggarwal Neelum T., Barnes Lisa L., Gallagher Aimee, Goldstein Jill M., Kantarci Kejal, Mallampalli Monica P., Mormino Elizabeth C., Scott Laura, Yu Wai Haung, Maki Pauline M., Mielke Michelle M. (2018). Understanding the impact of sex and gender in Alzheimer's disease: A call to action. Alzheimer's & Dementia.

[11] Pike Christian J. (2001). Estrogen Modulates Neuronal Bcl-xl Expression and β-Amyloid-Induced Apoptosis. Journal of Neurochemistry.

[12] Neu Scott C., Pa Judy, Kukull Walter, Beekly Duane, Kuzma Amanda, Gangadharan Prabhakaran, Wang Li-San, Romero Klaus, Arneric Stephen P., Redolfi Alberto, Orlandi Daniele, Frisoni Giovanni B., Au Rhoda, Devine Sherral, Auerbach Sanford, Espinosa Ana, Boada Mercè, Ruiz Agustín, Johnson Sterling C., Koscik Rebecca, Wang Jiun-Jie, Hsu Wen-Chuin, Chen Yao-Liang, Toga Arthur W. (2017). Apolipoprotein E Genotype and Sex Risk Factors for Alzheimer Disease. JAMA Neurology.

[13] Mangold CA, Wronowski B, Du M, Masser DR, Hadad N, Bixler GV, et al. Sexually divergent induction of microglial-associated neuroinflammation with hippocampal aging. J Neuroinflammation. 2017. 10.1186/s12974-017-0920-8. 10.1186/s12974-017-0920-8 PMC5521082 28732515

[14] Zhao Liqin, Mao Zisu, Woody Sarah K., Brinton Roberta D. (2016). Sex differences in metabolic aging of the brain: insights into female susceptibility to Alzheimer's disease. Neurobiology of Aging.

[15] Wixted John, Cai Denise J. (2013). Memory Consolidation.

[16] Braak H., Braak E. (1991). Neuropathological stageing of Alzheimer-related changes. Acta Neuropathologica.

[17] Caldwell Jessica Z.K., Berg Jody-Lynn, Shan Guogen, Cummings Jeffrey L., Banks Sarah J. (2018). Sex Moderates the Impact of Diagnosis and Amyloid PET Positivity on Hippocampal Subfield Volume. Journal of Alzheimer's Disease.

[18] Borland Emma, Nägga Katarina, Nilsson Peter M., Minthon Lennart, Nilsson Erik D., Palmqvist Sebastian (2017). The Montreal Cognitive Assessment: Normative Data from a Large Swedish Population-Based Cohort. Journal of Alzheimer's Disease.

[19] Huisman Martijn, Kunst Anton E., Mackenbach Johan P. (2003). Socioeconomic inequalities in morbidity among the elderly; a European overview. Social Science & Medicine.

[20] The Swedish BIOFINDER Study. Available from: http://biofinder.se. Accessed 19 Oct 2018.

[21] Folstein Marshal F., Folstein Susan E., McHugh Paul R. (1975). “Mini-mental state”. Journal of Psychiatric Research.

[22] American Psychiatric Association. Diagnostic and Statistical Manual of Mental Disorders. 5th ed. Washington DC: American Psychiatric Association; 2013.

[23] Palmqvist Sebastian, Zetterberg Henrik, Blennow Kaj, Vestberg Susanna, Andreasson Ulf, Brooks David J., Owenius Rikard, Hägerström Douglas, Wollmer Per, Minthon Lennart, Hansson Oskar (2014). Accuracy of Brain Amyloid Detection in Clinical Practice Using Cerebrospinal Fluid β-Amyloid 42. JAMA Neurology.

[24] Hansson Oskar, Seibyl John, Stomrud Erik, Zetterberg Henrik, Trojanowski John Q., Bittner Tobias, Lifke Valeria, Corradini Veronika, Eichenlaub Udo, Batrla Richard, Buck Katharina, Zink Katharina, Rabe Christina, Blennow Kaj, Shaw Leslie M. (2018). CSF biomarkers of Alzheimer's disease concord with amyloid-β PET and predict clinical progression: A study of fully automated immunoassays in BioFINDER and ADNI cohorts. Alzheimer's & Dementia.

[25] Palmqvist S, Schöll M, Strandberg O, Mattsson N, Stomrud E, Zetterberg H, et al. Earliest accumulation of β-amyloid occurs within the default-mode network and concurrently affects brain connectivity. Nat Commun. 2017. 10.1038/s41467-017-01150-x. 10.1038/s41467-017-01150-x PMC5663717 29089479

[26] Rosen WG, Mohs RC, Davis KL. A new rating scale for Alzheimer’s disease. Am J Psychiatry. 1984. 10.1176/ajp.141.11.1356. 10.1176/ajp.141.11.1356 6496779

[27] TALLBERG I. M., IVACHOVA E., JONES TINGHAG K., ÖSTBERG P. (2008). Swedish norms for word fluency tests: FAS, animals and verbs. Scandinavian Journal of Psychology.

[28] Palmqvist Sebastian, Hansson Oskar, Minthon Lennart, Londos Elisabet (2008). The Usefulness of Cube Copying for Evaluating Treatment of Alzheimer's Disease. American Journal of Alzheimer's Disease & Other Dementiasr.

[29] Reitan Ralph M. (1955). The relation of the Trail Making Test to organic brain damage. Journal of Consulting Psychology.

[30] Fleisher AS, Sowell BB, Taylor C, Peterson RC, Thal LJ (2007). Clinical predictors of progression to Alzheimer’s disease in amnestic mild cognitive impairment.

[31] Jessen Frank, Amariglio Rebecca E., van Boxtel Martin, Breteler Monique, Ceccaldi Mathieu, Chételat Gaël, Dubois Bruno, Dufouil Carole, Ellis Kathryn A., van der Flier Wiesje M., Glodzik Lidia, van Harten Argonde C., de Leon Mony J., McHugh Pauline, Mielke Michelle M., Molinuevo Jose Luis, Mosconi Lisa, Osorio Ricardo S., Perrotin Audrey, Petersen Ronald C., Rabin Laura A., Rami Lorena, Reisberg Barry, Rentz Dorene M., Sachdev Perminder S., de la Sayette Vincent, Saykin Andrew J., Scheltens Philip, Shulman Melanie B., Slavin Melissa J., Sperling Reisa A., Stewart Robert, Uspenskaya Olga, Vellas Bruno, Visser Pieter Jelle, Wagner Michael (2014). A conceptual framework for research on subjective cognitive decline in preclinical Alzheimer's disease. Alzheimer's & Dementia.

[32] van Harten Argonde C., Visser Pieter Jelle, Pijnenburg Yolande A.L., Teunissen Charlotte E., Blankenstein Marinus A., Scheltens Philip, van der Flier Wiesje M. (2013). Cerebrospinal fluid Aβ42 is the best predictor of clinical progression in patients with subjective complaints. Alzheimer's & Dementia.

[33] Sundermann Erin E., Edmonds Emily C., Delano-Wood Lisa, Galasko Douglas R., Salmon David P., Rubin Leah H., Bondi Mark W. (2018). Sex Influences the Accuracy of Subjective Memory Complaint Reporting in Older Adults. Journal of Alzheimer's Disease.

[34] Buckley RF, Villemagne VL, Masters CL, Ellis KA, Rowe CC, Johnson KA, et al. A conceptualization of the utility of subjective cognitive decline in clinical trials of preclinical Alzheimer’s disease. J Mol Neurosci. 2016. 10.1017/S0954579414000868.Child-evoked. 10.1007/s12031-016-0810-z PMC5241130 27514526

[35] Krogsrud Stine K., Tamnes Christian K., Fjell Anders M., Amlien Inge, Grydeland Håkon, Sulutvedt Unni, Due-Tønnessen Paulina, Bjørnerud Atle, Sølsnes Anne E., Håberg Asta K., Skrane Jon, Walhovd Kristine B. (2014). Development of hippocampal subfield volumes from 4 to 22 years. Human Brain Mapping.

[36] Ardekani Babak A., Convit Antonio, Bachman Alvin H. (2016). Analysis of the MIRIAD Data Shows Sex Differences in Hippocampal Atrophy Progression. Journal of Alzheimer's Disease.

[37] Nosheny Rachel L., Insel Philip S., Truran Diana, Schuff Norbert, Jack Clifford R., Aisen Paul S., Shaw Leslie M., Trojanowski John Q., Weiner Michael W. (2015). Variables associated with hippocampal atrophy rate in normal aging and mild cognitive impairment. Neurobiology of Aging.

[38] Marrocco J, McEwen BS. Sex in the brain: Hormones and sex differences. Dialogues Clin Neurosci. 2016. 10.12032/TMR201705034. 10.31887/DCNS.2016.18.4/jmarrocco PMC5286723 28179809

[39] Buckley Rachel F., Mormino Elizabeth C., Chhatwal Jasmeer, Schultz Aaron P., Rabin Jennifer S., Rentz Dorene M., Acar Diler, Properzi Michael J., Dumurgier Julien, Jacobs Heidi, Gomez-Isla Teresa, Johnson Keith A., Sperling Reisa A., Hanseeuw Bernard J. (2019). Associations between baseline amyloid, sex, and APOE on subsequent tau accumulation in cerebrospinal fluid. Neurobiology of Aging.

[40] Riedel Brandalyn C., Thompson Paul M., Brinton Roberta Diaz (2016). Age, APOE and sex: Triad of risk of Alzheimer’s disease. The Journal of Steroid Biochemistry and Molecular Biology.

