# Supplementary figures and images for: Cognitively normal women with Alzheimer’s disease proteinopathy show relative preservation of memory but not of hippocampal volume
Source: Alzheimers Res Ther. 2019 Dec 26;11:109. doi: 10.1186/s13195-019-0565-1 (PMC6933621; doi:10.1186/s13195-019-0565-1)

## A. Subjective cognitive decline    B. Mild cognitive impairment

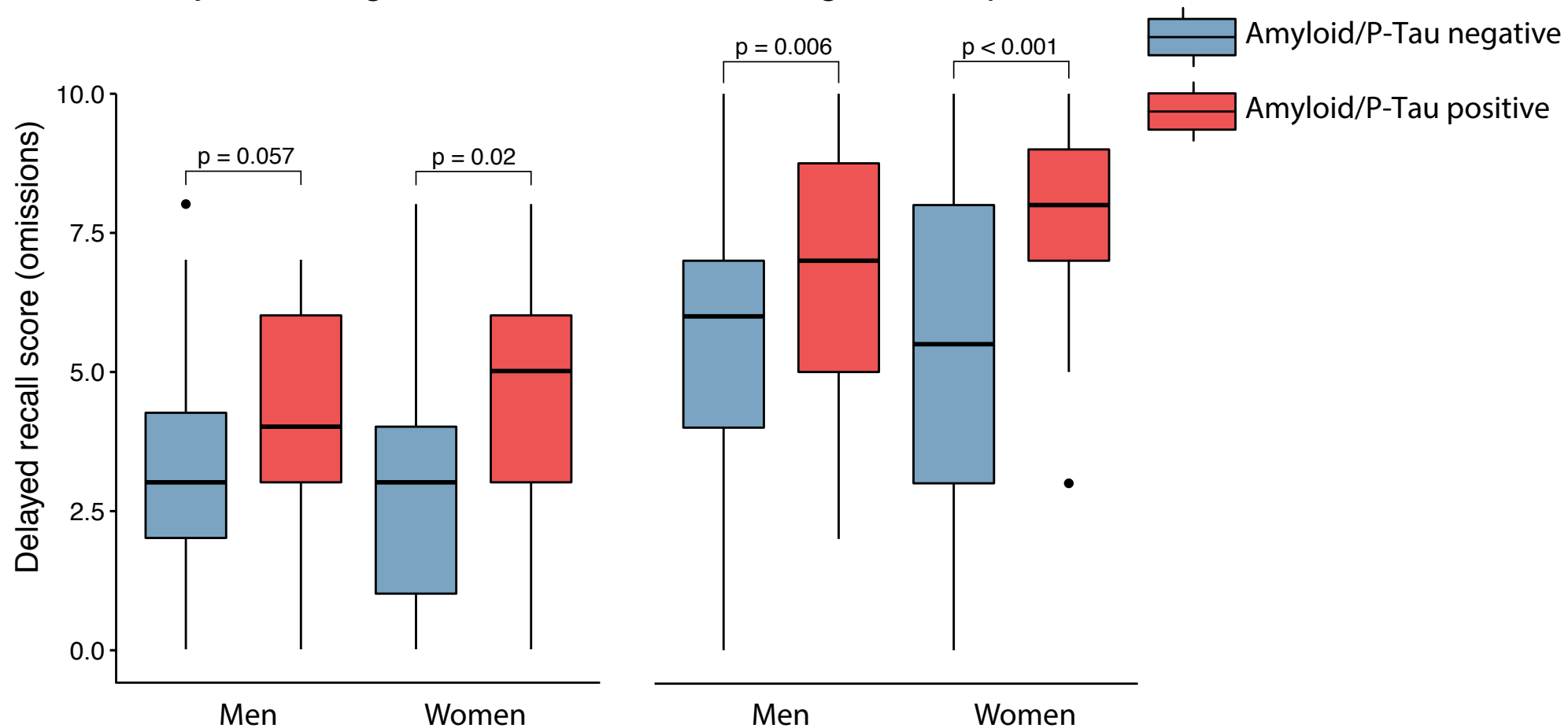

Supplement: Supplementary file 1 — Additional file 1: Figure S1. Comparison of the Aβ/P-tau effect on memory in men and women. Comparison of 10 word delayed recall performance in Aβ/P-tau+ and Aβ/P-tau- participants, stratified by sex, in patients with A. subjective cognitive decline, and B. mild cognitive symptoms. [file 13195_2019_565_MOESM1_ESM.pdf]
