# Supplementary material for: Cognitively normal women with Alzheimer’s disease proteinopathy show relative preservation of memory but not of hippocampal volume
Source: Alzheimers Res Ther. 2019 Dec 26;11:109. doi: 10.1186/s13195-019-0565-1 (PMC6933621; doi:10.1186/s13195-019-0565-1)
Supplement: Supplementary file 2 — Additional file 2: Regression Predicting Hippocampal Volumes with Cognitively Normal, Subjective Cognitive Decline, and Mild Cognitive Impairment Groups. This additional file contains a table summarizing the results of a regression predicting hippocampal volumes separated by diagnostic group. [file 13195_2019_565_MOESM2_ESM.docx]

| **Additional File 1** | | | |  |  |  |  |
| --- | --- | --- | --- | --- | --- | --- | --- |
|  | **Left Hippocampus** | | | | |  |  |
| Variable | Estimate (CI) | | *p* | |  |  |  |
|  |  | |  | |  |  |  |
| Intercept | 5514.76 (4730.41 − 6299.11) | | **<0.001** | |  |  |  |
|  |  | |  | |  |  |  |
| Diagnosis  (1=CN, 2=SCD, 3=MCI) | -233.23 (−311.86 − -154.60) | | **<0.001** | |  |  |  |
|  |  | |  | |  |  |  |
| Age | -43.03 (−50.17 − -35.89) | | **<0.001** | |  |  |  |
|  |  | |  | |  |  |  |
| Education | -2.19 (−12.99 − 8.61) | | 0.692 | |  |  |  |
|  |  | |  | |  |  |  |
| Total Intracranial Volume (z-scored) | 0.27 (0.19 − 0.35) | | **<0.001** | |  |  |  |
|  |  | |  | |  |  |  |
| Sex (0=male) | -53.86 (−287.91 − 180.18 | | 0.652 | |  |  |  |
|  |  | |  | |  |  |  |
| APOE ε4 Genotype  (0=no ε4) | 21.78 (−64.12 − 107.68) | | 0.619 | |  |  |  |
|  |  | |  | |  |  |  |
| CSF Aβ/P-tau Positivity (Aβ/P-tau+) (0=not Aβ/P-tau+) | -22.60 (−384.07− 338.87) | | 0.902 | |  |  |  |
|  |  | |  | |  |  |  |
| Aβ/P-tau+ x Diagnosis | -39.12 (−184.63 − 106.38) | | 0.598 | |  |  |  |
|  |  | |  | |  |  |  |
| Aβ/P-tau+ x Sex | 42.3 (−427.6 − 512.2) | | 0.86 | |  |  |  |
|  |  | |  | |  |  |  |
| Diagnosis x Sex | -6.84 (−124.35 − 110.67) | | 0.909 | |  |  |  |
|  |  | |  | |  |  |  |
| Aβ/P-tau+ x Diagnosis x Sex | -42.24 (−243.47 − 159) | | 0.681 | |  |  |  |
|  |  |  |  | |  |  |  |
|  | **Right Hippocampus** | | | | |  |  |
| Variable | Estimate (CI) | | *p* | |  |  |  |
|  |  |  |  | |  |  |  |
| Intercept | 5388.66 (4584.86 − 6192.47) | | **<0.001** | |  |  |  |
|  |  | |  | |  |  |  |
| Diagnosis  (1=CN, 2=SCD, 3=MCI) | -196.57 (−277.15 − -115.99) | | **<0.001** | |  |  |  |
|  |  | |  | |  |  |  |
| Age | -41.93 (−49.25 − -34.62) | | **<0.001** | |  |  |  |
|  |  | |  | |  |  |  |
| Education | -1.15 (−12.22 − 9.92) | | 0.839 | |  |  |  |
|  |  | |  | |  |  |  |
| Total Intracranial Volume (z-scored) | 0.28 (0.20 − 0.36) | | **<0.001** | |  |  |  |
|  |  | |  | |  |  |  |
| Sex (0=male) | -56.27 (−296.13 − 183.58) | | 0.646 | |  |  |  |
|  |  | |  | |  |  |  |
| APOE ε4 Genotype  (0=no ε4) | 15.1 (−72.93 − 103.14) | | 0.737 | |  |  |  |
|  |  | |  | |  |  |  |
| CSF Aβ/P-tau Positivity (Aβ/P-tau+) (0=not Aβ/P-tau+) | 183.42 (−187.01 − 553.86) | | 0.332 | |  |  |  |
|  |  | |  | |  |  |  |
| Aβ/P-tau+ x Diagnosis | -119.49 (−268.6 − 29.63) | | 0.117 | |  |  |  |
|  |  | |  | |  |  |  |
| Aβ/P-tau+ x Sex | -103.31 (−584.87 − 378.24) | | 0.674 | |  |  |  |
|  |  | |  | |  |  |  |
| Diagnosis x Sex | 14.75 (−105.68 − 135.17) | | 0.81 | |  |  |  |
|  |  | |  | |  |  |  |
| Aβ/P-tau+ x Diagnosis x Sex | 19.33 (−186.9 − 225.55) | | 0.854 | |  |  |  |
|  |  |  |  | |  |  |  |
|  | **Total Hippocampal Volume** | | | | |  |  |
| Variable | Estimate (CI) | | *p* | |  |  |  |
|  |  | |  | |  |  |  |
| Intercept | 10903.42 (9415.43 − 12391.41) | | **<0.001** | |  |  |  |
|  |  | |  | |  |  |  |
| Diagnosis  (1=CN, 2=SCD, 3=MCI) | -429.80 (−578.97 − -280.64) | | **<0.001** | |  |  |  |
|  |  | |  | |  |  |  |
| Age | -84.96 (−98.51 − -71.42) | | **<0.001** | |  |  |  |
|  |  | |  | |  |  |  |
| Education | -3.34 (−23.83 − 17.15) | | 0.75 | |  |  |  |
|  |  | |  | |  |  |  |
| Total Intracranial Volume (z-scored) | 0.0021 (0.21 − 0.37) | | **<0.001** | |  |  |  |
|  |  | |  | |  |  |  |
| Sex (0=male) | -110.14 (−554.15 − 333.88) | | 0.627 | |  |  |  |
|  |  | |  | |  |  |  |
| APOE ε4 Genotype  (0=no ε4) | 36.88 (−126.08 − 199.85) | | 0.657 | |  |  |  |
|  |  | |  | |  |  |  |
| CSF Aβ/P-tau Positivity (Aβ/P-tau+) (0=not Aβ/P-tau+) | 160.82 (−524.92 − 846.56) | | 0.646 | |  |  |  |
|  |  | |  | |  |  |  |
| Aβ/P-tau+ x Diagnosis | -158.61 (−434.65 − 117.43) | | 0.261 | |  |  |  |
|  |  | |  | |  |  |  |
| Aβ/P-tau+ x Sex | -61.02 (−952.46 − 830.43) | | 0.893 | |  |  |  |
|  |  | |  | |  |  |  |
| Diagnosis x Sex | 7.91 (−215.01 − 230.83) | | 0.945 | |  |  |  |
|  |  | |  | |  |  |  |
| Aβ/P-tau+ x Diagnosis x Sex | -22.91 (−404.68 − 358.85) | | 0.906 | |  |  |  |
|  |  |  |  | |  |  |  |
| *NOTE Estimates, confidence intervals, and *p* values were calculated for each regression model. | | | | | | | |

| †Abbreviations: Aβ/P-tau+, amyloid beta/P-tau positivity; APOE, Apolipoprotein E; CSF, cerebrospinal fluid |  |  |  |
| --- | --- | --- | --- |
